# Cornulin as a Prognosticator for Lymph Node Involvement in Cutaneous Squamous Cell Carcinoma

**DOI:** 10.7759/cureus.33130

**Published:** 2022-12-30

**Authors:** Rachna Karumuri, Dean Shah, Hilal Arnouk

**Affiliations:** 1 Osteopathic Medicine, Midwestern University Chicago College of Osteopathic Medicine, Downers Grove, USA; 2 Pathology, Midwestern University Chicago College of Osteopathic Medicine, Downers Grove, USA

**Keywords:** lymph node metastasis, nodal status, tnm classification, cornulin, cutaneous squamous cell carcinoma (scc), nonmelanoma skin cancer, immunohistochemistry (ihc), computer-assisted image analysis

## Abstract

Background

Cornulin is an epidermal differentiation marker and a stress-related protein. Its expression patterns are likely to reflect the multi-step tumorigenesis process of cSCC, given its role as a tumor suppressor. The aim of this study is to evaluate the utility of Cornulin as a prognosticator for cutaneous squamous cell carcinoma (cSCC). Specifically, the correlation between Cornulin expression and the clinicopathological parameter of lymph node involvement (nodal status), which plays a major role in determining cSCC prognosis and recurrence. We predicted that Cornulin expression declines as cSCC tumors metastasize to regional lymph nodes.

Methodology

Tissue samples of cSCC lesions of variable nodal involvement status were stained using immunohistochemistry, and high-resolution images were acquired. Aperio ImageScope software (Leica Biosystems) equipped with a positive-pixel-counting algorithm was used to quantify the staining intensity. Subsequently, Cornulin immunoreactivity was calculated as a Histo-score (H-score) value, which is based on the staining intensity and the percentage of positively stained cells. Mean H-scores were compared between groups using an unpaired t-test.

Results

A significant inverse correlation was found between Cornulin expression levels and metastasis to the lymph nodes. Specifically, primary tumors with metastasis to regional lymph nodes (N1) exhibited 9.5-fold decrease in Cornulin immunoreactivity compared to the primary tumor samples without lymph node involvement (N0).

Conclusion

Cornulin was found to be significantly downregulated in primary tumors with lymph node metastases. Detection assays to measure Cornulin expression in cSCC primary tumors might aid in determining the nodal status in these patients and possibly help determine cases of occult lymph node metastasis or micrometastasis. Future clinical studies are needed to help establish Cornulin’s role in enhancing the predictive power of histopathological examination and improving survival rates for patients suffering from this type of skin cancer.

## Introduction

Cornulin is a squamous cell-specific protein that has recently been associated with several human disease processes. Encoded by the CRNN gene located on chromosome 1q21 within the epidermal differentiation complex fused-gene complex, Cornulin is highly expressed in mature keratinocytes, especially in the granular and lower cornified cell layers of the epidermis [1-3]. Therefore, it is considered to be a late epidermal differentiation marker [4]. Cornulin is also known as squamous epithelial heat shock protein 53 (SEP53) due to its participation in heat shock and stress responses in the esophageal squamous epithelium [5,6]. Moreover, SEP53 response to stress is linked to its activity as a survival factor. For instance, SEP53 can attenuate apoptotic cell death induced by lethal levels of deoxycholic acid caused by acid and bile reflux in the upper gastrointestinal tract [7]. Interestingly, Cornulin is found to be upregulated in psoriatic hyperproliferative skin lesions in response to inflammatory cytokines, where it has been shown to positively regulate keratinocyte proliferation through the activation of phosphoinositide 3-kinase (PI3K) and Akt pathway [8]. On the other hand, Cornulin overexpression in oral and esophageal squamous cell carcinoma cell lines leads to cell cycle arrest at the G1/S checkpoint and downregulation of cyclin D1 expression [9,10], perhaps highlighting its role as tumor suppressor gene product [10].

While Cornulin is found to be upregulated in acute stress responses, it is notably downregulated in several malignancies involving the squamous cell epithelium. Studies have documented this downward trend in Cornulin expression in cervical SCC [11,12], oral SCC [13-15], esophageal SCC [6,16], and cutaneous SCC [17].

Given that cutaneous SCC (cSCC) is a relatively common skin cancer that can metastasize and recur, we aimed to characterize Cornulin as a molecular biomarker that can potentially aid in monitoring disease progression and relapse, as well as predicting clinical outcomes for cSCC patients, by examining the correlation between Cornulin expression and the clinical stages as indicated by the TNM staging of cSCC based on tumor size, regional lymph node involvement, and metastasis. Our findings suggest that Cornulin might serve as an indicator for lymph node involvement status in cases of cutaneous squamous cell carcinoma.

## Materials and methods

Immunohistochemistry staining of tissue samples

The tissue samples for this experiment were obtained from de-identified tissue microarrays (US Biomax and US Biolabs, Rockville, MD) and included 16 primary tumors from head and neck, trunk and extremities sites. Eight primary tumors were associated with no lymph node metastasis (N0), and eight primary tumors were associated with lymph node metastasis (N1). Additionally, serial sections of a normal skin tissue sample were used as equalizing control in each IHC experiment. During the immunohistochemistry staining and analysis, the experimenters were blinded to the TNM classification and clinical stages of the tissue samples.

Immunohistochemistry staining of tissue microarray slides consisted of an antigen retrieval step using citric acid followed by blocking with 5% bovine serum albumin (BSA). The tissue samples were then labeled with a rabbit IgG polyclonal primary antibody against Cornulin at 1:200 dilution (Sigma HPA024343; Sigma-Aldrich, St. Louis, MO), followed by staining with anti-rabbit HRP-conjugated secondary antibody at 1:1000 dilution (Sigma A9169; Sigma-Aldrich, St. Louis, MO). The tissue samples were incubated with 3,3' Diaminobenzidine (DAB) chromogenic substrate and counterstained with Hematoxylin, then mounted with a coverslip for preservation.

Quantitative analysis of Cornulin expression

Following the IHC experiment protocol, the tissue samples were imaged with a high-resolution Nikon A1R (Tokyo, Japan) inverted microscope at 10x magnification. Aperio ImageScope software (Leica Biosystems Inc., Buffalo Grove, IL) was used for computer-assisted image analysis to eliminate interobserver bias and subjectivity that is often seen in the manual assessment of IHC staining intensity. Quantification of immunoreactivity in the tissue region of interest was accomplished using the positive pixel counting v9 algorithm in Aperio ImageScope software. Manual selection of regions of interest (i.e., tumor parenchyma) in each tissue sample was included, while irrelevant regions were excluded (i.e., dermis and stroma). Strong (Nsp), medium (Np), weak (Nwp) positive pixels, and negative pixels were assigned red, orange, yellow, and blue colors, respectively, by the Aperio ImageScope (Sausalito, CA) software. The algorithm generated the number of Nwp, Np, and Nsp positive pixels; the total number of positive and negative pixels (NTotal); and the percentage of total positivity (NPositive/NTotal), which allowed for the calculation of the percentage of the weak, medium, and strong positivity by dividing Nwp, Np, and Nsp pixels, respectively, by NTotal pixels. Immunoreactivity was measured based on two parameters, the percentage of positively stained epithelial cells and the staining intensity. Histo-score (H-score) was calculated by adding the percentage of positive cells multiplied by the weighted intensity of staining. H-score = (1 x % weak positivity) + (2 x % medium positivity) + (3 x % strong positivity). The percentage of positive cells (% staining) was derived from the percentage of total positivity (NPositive/NTotal) values produced by the Aperio ImageScope software. The average H-scores for each set of clinicopathological parameters were calculated along with the standard error of the mean (SEM), followed by statistical analysis using a two-sample unpaired t-test.

## Results

Cornulin expression in cutaneous squamous cell carcinoma

**Figure 1:**
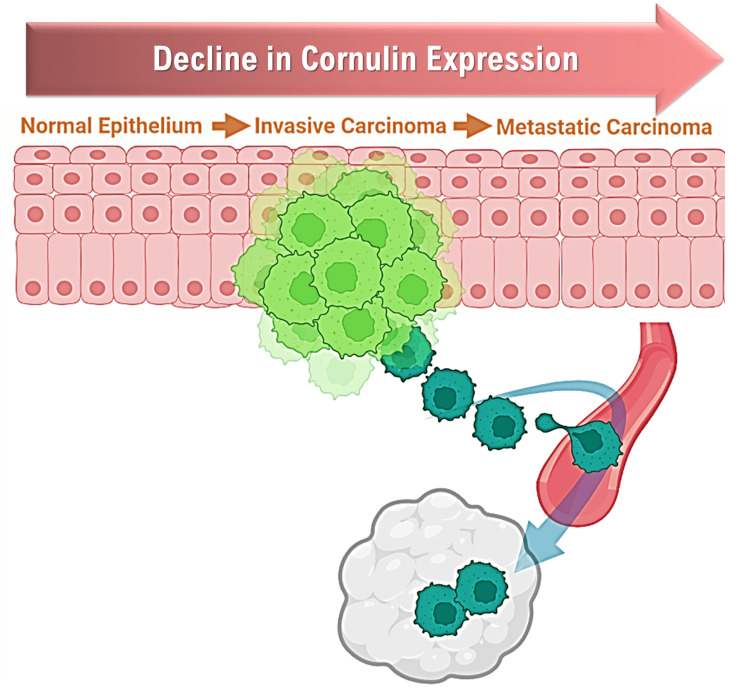
Expected trends in Cornulin expression with the progression of cutaneous squamous cell carcinoma. Schematic graph showing a predicted decline in Cornulin expression along the progression steps of cutaneous squamous cell carcinoma. This original figure was drawn by authors using Microsoft Office PowerPoint (Redmond, USA) and BioRender software.

As a late epidermal differentiation marker, Cornulin is abundant in the upper layers of the epidermis [4]. We have established in an earlier study that Cornulin is less expressed, or even lost, in cutaneous squamous cell carcinoma tissue samples [17]. In this study, we investigated the potential correlation between Cornulin expression and cSCC tumor progression as measured by the different TNM clinicopathological parameters, especially the correlation to the nodal status due to the significant detrimental effect of lymph node involvement on prognosis in patients with this type of skin cancer [18-22] (Figure 1).

Correlation between Cornulin expression and lymph node involvement in cSCC

**Figure 2:**
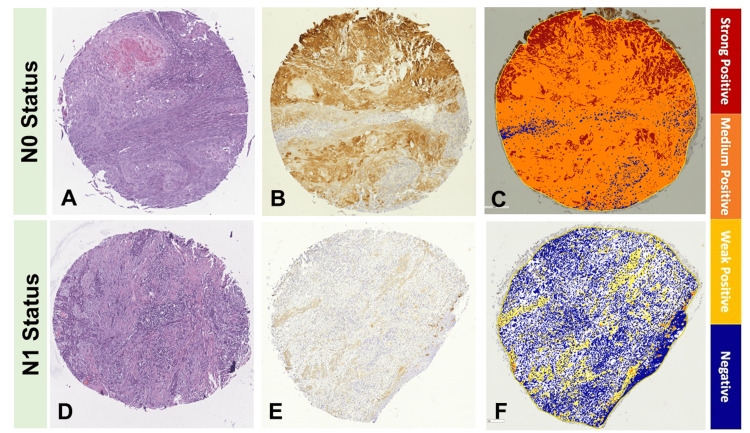
Cornulin expression in cutaneous squamous cell carcinoma tissue samples with variable nodal status. The upper panel shows a representative cSCC tissue sample from a primary tumor associated with N0 status: (A) H&E stained, (B) IHC-stained with Cornulin, (C) Image scope analyzed. The lower panel shows a representative cSCC tissue sample from a primary tumor associated with N1 status: (D) H&E stained, (E) IHC-stained with Cornulin, (F) Image scope analyzed.

Cornulin immunohistochemical detection combined with computer-assisted image analysis showed that tissue samples with metastases to the lymph nodes, N1, exhibit a significant decline in Cornulin expression when compared to tissue samples without nodal metastases, N0 (Figure 2).

**Figure 3:**
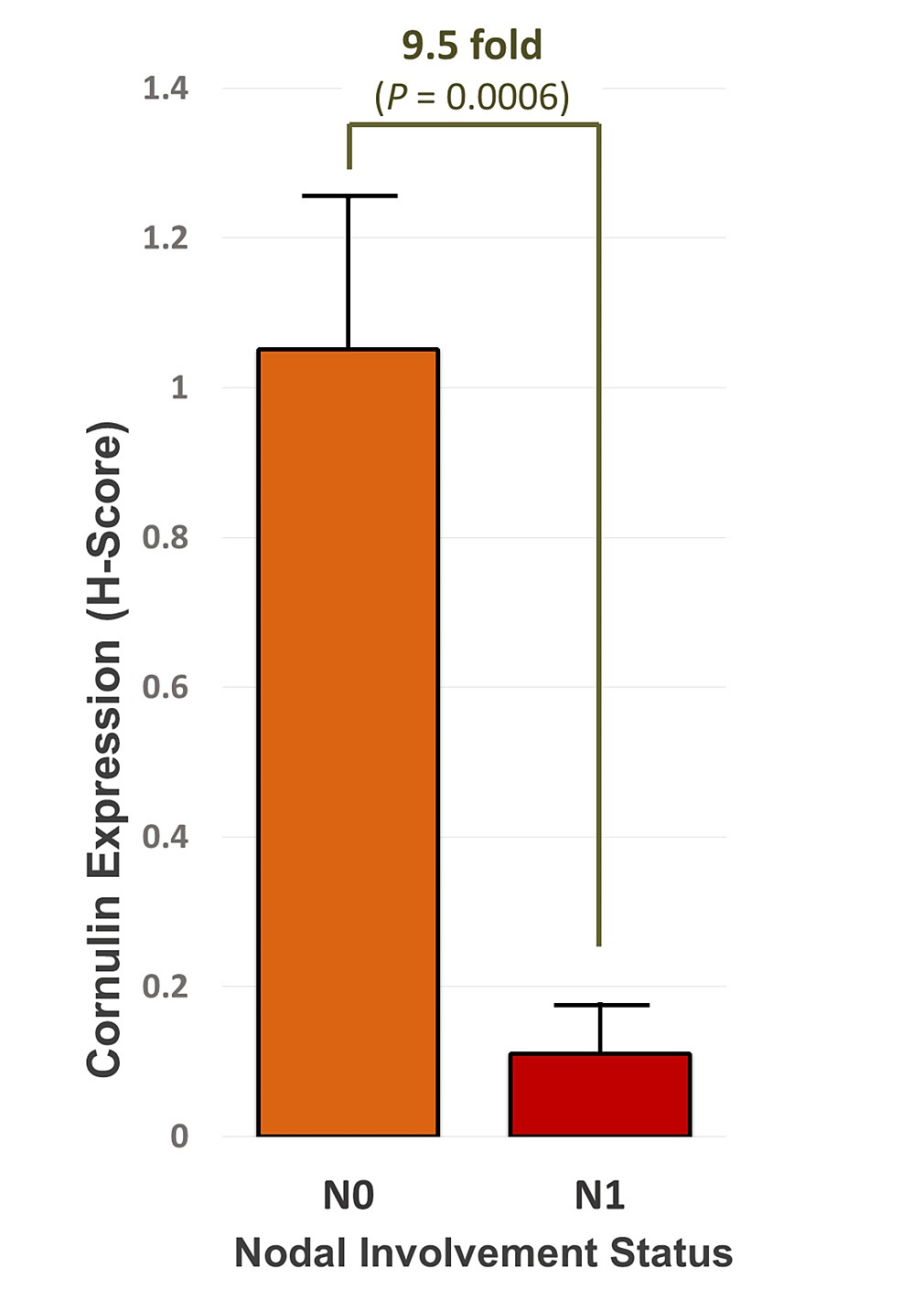
Correlation between Cornulin expression and nodal involvement status in cutaneous squamous cell carcinoma tissue samples. Bar graph depicting mean H-scores for cornulin immunoreactivity in eight cSCC tissue samples from a primary tumor associated with no lymph node metastases (N0) and eight cSCC tissue samples from a primary tumor associated with lymph node metastases (N1).

The mean H-score for Cornulin immunoreactivity was 1.05 for samples with N0 status and 0.11 for samples with N1 status. On average, there was a 9.5-fold downregulation in Cornulin expression associated with the N1 tissue samples. The correlation between Cornulin expression and the nodal status of cSCC was statistically significant (P = 0.0006) (Figure 3).

## Discussion

Cutaneous squamous cell carcinoma is the second most common malignant tumor of the skin in White patients and the most common malignant skin tumor in Black patients [23]. Importantly, the incidence of cSCC has also been increasing in recent years due to various factors, such as lifetime exposure to ultraviolet radiation through tanning, occupational exposure, or through the decreasing ozone layer due to climate change [24-26]. Additionally, cSCC carries the risk of metastasis to regional lymph nodes when it escapes early detection. Interestingly, targeted therapies for metastatic melanoma using BRAF inhibitors, such as vemurafenib and dabrafenib that block the active conformation of the BRAF kinase, are associated with a higher risk of cSCC development [27,28]. The rising incidence of cSCC warrants the pursuit of novel biomarker prognosticators that can complement current histopathological methods and augment the accuracy of diagnostic and prognostic tests for cutaneous squamous cell carcinoma.

Currently, the evaluation of prognosis in cSCC patients is heavily reliant on the TNM classification to assess the tumor size (T), regional lymph node involvement (N), and distant metastases (M) parameters. However, patients with similar TNM staging classification often have different clinical outcomes, highlighting the fact that tumors that share the same TNM clinical staging at the time of diagnosis might have the variable potential for proliferation, invasiveness, and metastasis. Cutaneous squamous cell carcinoma tends to spread first to regional lymph nodes before it metastasizes to distal tissue sites [29]. Moreover, the mortality rate from cSCC is due, in large part, to uncontrolled regional recurrence, rather than distant organ metastasis [30], especially in African American patients who experience disproportionately higher metastatic and mortality rates than White, Latino, or Asian patients [23].

Given the fact that metastatic cSCC is associated with a high mortality rate (70%) and high recurrence rates (15%-28%), determining if a cSCC tumor has metastasized to regional lymph nodes is a crucial step in patient management [18-22]. Establishing the nodal status of cSCC accurately can help forecast the prognosis since these tumors tend to metastasize to regional lymph nodes while metastases to distant organs are rare [30].

In our study, we predicted that Cornulin expression patterns are likely to reflect the multi-step carcinogenesis process of cSCC, given its role as a tumor suppressor [10]. Indeed, a significant inverse correlation was found between Cornulin expression levels and metastasis to the lymph nodes. Specifically, primary tumors with metastasis to regional lymph nodes (N1) showed an approximately 10-fold decrease in Cornulin immunoreactivity compared to the tumor samples without lymph node involvement (N0). Based on these findings, we propose that Cornulin detection assays in cSCC primary tumors may be able to aid in determining lymph node involvement. Moreover, we envision that Cornulin might be used to determine occult lymph node metastasis, or micrometastasis, which are often missed using conventional staining methods and can lead to false negative results in cSCC patients [31]. Thus, improving the accuracy of histopathological evaluation and survival rates for patients suffering from this nonmelanoma skin cancer.

## Conclusions

Cornulin was found to be significantly downregulated in primary tumors with lymph node metastasis, a major contributor to cSCC progression to the advanced clinical stages. Altogether, Cornulin expression can potentially serve as a valuable prognosticator for cSCC patients as it correlates with metastasis to regional lymph nodes, a key factor in determining the prognosis and recurrence of cutaneous squamous cell carcinoma.
